# Genotypes of Hepatitis C Virus and Efficacy of Direct-Acting Antiviral Drugs among Chronic Hepatitis C Patients in a Tertiary Care Hospital

**DOI:** 10.3390/tropicalmed8020092

**Published:** 2023-01-30

**Authors:** Nahed Mohammed Hawsawi, Tamer Saber, Hussein M. Salama, Walaa S. Fouad, Howaida M. Hagag, Hayaa M. Alhuthali, Emad M. Eed, Taisir Saber, Khadiga A. Ismail, Hesham H. Al Qurashi, Samir Altowairqi, Mohmmad Samaha, Dalia El-Hossary

**Affiliations:** 1 Department of Clinical Laboratory Sciences, College of Applied Medical Sciences, Taif University, Taif 21944, Saudi Arabia; 2 Departments of Internal Medicine, Faculty of Medicine, Zagazig University, Zagazig 44519, Egypt; 3 Departments of Family Medicine, Faculty of Medicine, Zagazig University, Zagazig 44519, Egypt; 4 Department of Pathology, Faculty of Medicine, Al-Azhar University, Cairo 11884, Egypt; 5 Medical Microbiology and Immunology Department, Faculty of Medicine, Menoufia University, Shebinel Kom 32511, Egypt; 6 Department of Medical Microbiology and Immunology, Faculty of Medicine, Zagazig University, Zagazig 44519, Egypt; 7 Department of Parasitology, Faculty of Medicine, Ain Shams University, Cairo 11566, Egypt; 8 Gastroenterology and Hepatology Department, King Abdul-Aziz Specialized Hospital, Taif 26521, Saudi Arabia

**Keywords:** HCV, DAAs, sustained viral response, cirrhosis, HCV genotype

## Abstract

Hepatitis C virus (HCV) chronic infection is a major causative factor for several chronic liver diseases, including liver cirrhosis, liver cell failure, and hepatocellular carcinoma. The HCV has seven major genotypes. Genotype 4 is the most prevalent genotype in the Middle East, including Saudi Arabia, followed by genotype 1. The HCV genotype affects the response to different HCV treatments and the progression of liver disease. Currently, combinations of direct-acting antiviral drugs (DAAs) approved for the treatment of HCV achieve high cure rates with minimal adverse effects. Because real-world data from Saudi Arabia about the efficacy of DAAs are still limited, this study was conducted to assess the effectiveness of DAAs in treating patients with chronic hepatitis C and to identify the variables related to a sustained virologic response (SVR) in a real-world setting in Saudi Arabia. This prospective cohort study included 200 Saudi patients with chronic HCV who were 18 years of age or older and had been treated with DAAs at King Abdul-Aziz Specialized Hospital in Taif, Saudi Arabia, between September 2018 and March 2021. The response to treatment was assessed by whether or not an SVR had been achieved at week 12 post treatment (SVR12). An SVR12 was reached in 97.5% of patients. SVR12 rates were comparable for patients of different ages, between men and women, and between patients with and without cirrhosis. In addition, the SVR12 rates did not differ according to the infecting HCV genotype. In this study, the presence of cirrhosis and the patient’s gender were independent predictors of who would not reach an SVR12 (known here as the non-SVR12 group) according to the results of univariate and multivariate binary logistic regression analyses based on the determinants of SVR12. In this population of patients with chronic HCV infection, all DAA regimens achieved very high SVR12 rates. The patients’ gender and the presence of cirrhosis were independent factors of a poor response.

## 1. Introduction

Chronic infection with the hepatitis C virus (HCV) is a significant risk factor for developing cirrhosis, liver cell failure, and hepatocellular cancer [1]. In addition, extrahepatic manifestations can be experienced in approximately 40% to 70% of patients with chronic hepatitis C, including chronic kidney disease, metabolic syndrome, autoimmune diseases, lymphoproliferative disorders, and cardiovascular and central nervous system abnormalities [2,3]. Some chronic hepatitis C patients are asymptomatic until they develop severe complications. Delays in the diagnosis of chronic HCV infection can limit treatment options and increase the possibilities of adverse outcomes and the transmission of infection to others [4]. An estimated 58 million people are diagnosed with chronic hepatitis C yearly, and approximately 1.5 million new infections occur. HCV is transmitted parentally and mostly occurs owing to unsafe injection practices, unsafe health care, injection drug use, and sexual practices related to exposure to blood [5,6]. The overall reported prevalence rate of HCV in Saudi Arabia is approximately 1.2% [7]. The World Health Organization (WHO) has set a goal of a 90% decrease in new HCV infections and a 65% decrease in HCV-related deaths by 2030 [8].

HCV has a lipoprotein envelope and a 9.6 kb, single-stranded RNA genome. The genome encodes a large precursor polyprotein, which in turn is cleaved into various nonstructural proteins (p7, NS2, NS3, NS4A, NS4B, NS5A, and NS5B) and structural proteins (core, E1, and E2) [9,10]. The RNA polymerase of HCV has a high propensity for error during replication, leading to high rates of nucleotide substitution in the genome of the progeny virus particles and, consequently, vast genetic variability. Based on these genetic variations, HCV is divided into seven genotypes (GTs) (numbered from GT1 to GT7) and approximately 100 subgenotypes [11,12,13]. Globally, GT1 is the major genotype, followed by GT3, GT4, and GT2. GT 5 and GT6 cause the remaining HCV cases, i.e., approximately 5%. Additionally, four immigrants from Congo were diagnosed with GT7 in Canada. However, the distribution of HCV genotypes and subtypes is variable worldwide [14,15,16]. The most prevalent genotype in Saudi Arabia is GT4, followed by GT1 [17]. The HCV genotype affects the response to different HCV treatments, the progression of liver disease, and the overall prognosis of HCV disease [18]. Furthermore, knowledge of the distribution and patterns of HCV genotypes contributes to effective HCV infection control [19]. Therefore, in the context of HCV chronic infection, HCV genotype analysis should be seriously examined [20,21]. 

A sustained virologic response (SVR), which is defined as the absence of detectable HCV RNA for at least 12 weeks (SVR12) or 24 weeks (SVR24) after completing antiviral treatment, is the primary goal of treating chronic hepatitis C [22,23] Achieving an SVR significantly reduces the HCV-related hepatic and extrahepatic complications, enhances the quality of life, and prevents HCV transmission [24]. 

In the past, pegylated interferon-based therapy for a long duration (24 to 72 weeks) was the only option for people with chronic hepatitis C. This therapy was not ideal because it achieved an SVR in only a limited percentage of patients and caused many adverse side effects [25,26]. The chronic hepatitis C treatment landscape has changed significantly since 2011 with the development of direct-acting antiviral drugs (DAAs), which target viral replications. In 2014, a highly effective and well-tolerated second-generation DAA was introduced and is now considered the harbinger of a new era in the treatment of chronic hepatitis C [27]. Generally, the approved DAAs can be divided into three classes: NS3/NS4A protease inhibitors, which inhibit the processing of the HCV polyprotein; NS5A complex inhibitors inhibiting viral assembly; and NS5B polymerase inhibitors, which block HCV RNA replication. Each of these DAA classes includes several drugs, and currently, combinations from these approved three classes are recommended in clinical practice. These combinations enable high cure rates with minimal adverse effects, better tolerability, and a shorter treatment duration [22,28].

The efficacy of DAAs has been proven by several clinical trials with “ideal” patients, whose characteristics differed from those of patients using these medications in everyday clinical practice. Comorbidities and/or constitutional characteristics might reduce the effectiveness rates of DAAs reported in controlled clinical studies, suggesting that these findings may not reflect the real world [29,30]. Therefore, evaluations of these drugs in real-life settings from various parts of the world are critical. Because real-world data from Saudi Arabia are still limited, data collected from different ethnic groups are insufficient. This study was carried out to evaluate the efficacy of DAAs in treating chronic hepatitis C patients and to identify the factors associated with an SVR in a real-life experience from a large tertiary care hospital in Taif, Saudi Arabia. The study also aimed to report the frequency of HCV genotypes among the studied patients.

## 2. Materials and Methods

This prospective cohort, single-center study included 200 chronic HCV-infected Saudi patients (119 male and 81 female) who were 18 years of age or older and had been treated with DAAs at King Abdul-Aziz Specialized Hospital, a large tertiary care hospital in Taif, Saudi Arabia, between September 2018 and March 2021. 

**Inclusion criteria:** This study included both treatment-naïve patients (individuals who had never been treated for HCV before) and treatment-experienced patients (those who had been treated before).

- A patient who met the following criteria was eligible for the study:
  1. Positive HCV-RNA;
  2. Age ≥ 18 years.
- Exclusion criteria: the following patients were excluded from the study:
  1. Pregnant or breastfeeding women;
  2. Decompensated cirrhosis (Child–Pugh C patients);
  3. Patient with hepatocellular carcinoma;
  4. Patient with systemic malignancy except after 2 years of disease-free interval;
  5. Patients with concurrent hepatitis B virus and/or HIV infection;
  6. Platelet count less than 500,000/mm^3^;
  7. Patients with significant illnesses such as congestive heart failure, renal failure, respiratory failure, or autoimmune diseases.

All patients were subjected to full history taking and clinical examination, abdominal ultrasonography, and routine laboratory tests, including complete blood count, international normalization ratio (INR), partial thromboplastin time (PTT), serum creatinine, serum albumin, total serum bilirubin, alanine aminotransferase (ALT), and alkaline phosphatase (ALP); gamma-glutamyl transferase (GT) and alfa fetoprotein (AFP), which were taken at the beginning of treatment (basal) and again 12 weeks after treatment ended); and HCV genotype, which was performed only at baseline. Child–Turcotte–Pugh score was calculated for each patient.

**Assessment of fibrosis:** The degree of fibrosis and cirrhosis was assessed using transient elastography (FibroScan): Assessment of liver stiffness using vibration controlled transient elastography is a favored and non-invasive modality. For staging of liver fibrosis, the cut-off values were F1 (>4.8) and (≤7.0 kPa), and F2 ranged between 7.0 and 9.5 kPa, while F3 equal or higher than 9.5 kPa and less than 12.0 kPa and F4 was considered if reading was ≥12.0 kPa. The XL probe was used for the examination of obese patients [31].

Both individuals who had never been treated for HCV before (treatment-naïve) and those who had been treated before (treatment-experienced) participated in the trial. Pregnant or breastfeeding women; patients with significant illnesses such as congestive heart failure, renal failure, respiratory failure, or autoimmune diseases; and patients with concurrent hepatitis B virus and/or HIV infection were excluded from this research. To assess cirrhosis and associated issues, all patients were subjected to full history taking and clinical examination in addition to a baseline abdominal ultrasound and routine laboratory investigation. The grade of liver fibrosis was assessed by using transient elastography (TE) with FibroScan.

These lab tests were done at the beginning of treatment and again 12 weeks after treatment ended: complete blood count, international normalization ratio (INR), partial thromboplastin time (PTT), serum creatinine, serum albumin, total serum bilirubin, alanine aminotransferase (ALT), alkaline phosphatase (ALP), and gamma-glutamyl transferase (-GT). HCV genotype was performed only at baseline.

Study participants were regularly monitored for 12 weeks after therapy ended. The research Ethics Committee of Taif University and King Abdul-Aziz Specialized Hospital approved this study, and each participating patient signed a written informed consent form. 

### 2.1. Screening for HCV Infection

According to the manufacturer’s instructions, patients were screened for HCV infection by MonolisaTM HCV Ag-Ab ULTRA V2 (BIO-RAD France). This assay is a qualitative enzyme immunoassay for the detection of anti-HCV antibodies and HCV capsid antigen in human serum or plasma. It uses two recombinant proteins from the non-structural region (NS3 and NS4) and a peptide from the structural region (capsid) of the HCV for the detection of the anti-HCV antibodies. A monoclonal antibody against the HCV capsid is used to detect the HCV capsid antigen.

### 2.2. Confirmation of HCV Infection and Quantification of HCV RNA

Identification of HCV infection was confirmed by quantitative HCV- RNA PCR using COBAS^®^ AmpliPrep/COBAS^®^ TaqMan^®^ HCV Quantitative Test, version 2.0 (lower limit of detection, 15 IU/mL), according to the manufacturer’s instructions. This test is used for quantifying HCV RNA genotypes 1 to 6 in human EDTA plasma or serum using the COBAS^®^ AmpliPrep Instrument for automated specimen processing and the COBAS^®^ TaqMan^®^ Analyzer or the COBAS^®^ TaqMan^®^ 48 Analyzer for automated amplification and detection of HCV RNA. 

### 2.3. Determination of HCV Genotype

HCV genotype was determined by the FDA-approved Abbott RealTime HCV Genotype II assay (Abbott Molecular Inc., Des Plaines, IL, USA), according to the manufacturer’s instructions. These clinical samples are processed by an RT-PCR technique that amplifies the RNA genome of HCV. In addition, at the outset of sample processing, a different RNA sequence than the HCV target sequence is injected into each specimen. For each sample, this unrelated RNA sequence is amplified alongside the target mRNA, using RT-PCR to ensure that the amplification process went well. The assay detects genotypes 1–6 and subtypes 1a and 1b using genotype-specific, fluorescent-labelled oligonucleotide probes.

### 2.4. The Treatment Decision

According to the AASLD-IDSA and Saudi Association for the Study of Liver Disease (SASLT) recommendations for hepatitis C, the attending physicians chose the treatment plan [31,32]. Patients were treated with one of the following treatment regimens:

1. Sofosbuvir/Daclatasvir ± Ribavirin (SOF/DCV 400/60 mg once daily oral dose ± RBV) as a pangenomic regimen for 12 weeks for non-cirrhotic and compensated cirrhotic patients;
2. Sofosbuvir/Ledipasvir ± Ribavirin (SOF/LDV 400/90 mg once daily dose ± RBV) for GT1 for 8 or 12 weeks according to the baseline HCV RNA and the cirrhosis state;
3. Ombitasvir/Paritaprevir/Ritonavir/Dasabuvir ± Ribavirin (OBV/PTV/Rtv (25/150/100 mg once daily orally) plus DSV (250 mg twice daily orally) ± RBV) for 12 weeks for non-cirrhotic GT 1a patients;
4. Elbasvir/Grazoprevir (EBR/GZR) 50/100 mg single oral daily dose for 12 weeks as a non-pangenomic regimen for patients with GT1b;
5. Glecaprevir/Pibrentasvir/Ribavirin (GLE/PIB 300/120 mg single daily oral dose ± RBV) as a pangenotypic regimen for 8 weeks.

The medication regimen was selected depending on genotypes, treatment history, and the presence or absence of cirrhosis. The dose of RBV for non-cirrhotic or CTP A was weight-based: 1200 mg (for patients ≥ 75 Kg) given orally daily (in two divided doses), while those >75 Kg body weight received 1000 mg orally daily (in two divided doses). For cirrhotic CTP B receiving SOF/LDV, the dose was RBV 600 mg/day, which increased by 200 mg/day every 2 weeks as tolerated. For patients with renal impairment, CrCl 30–50 mL/min 200 mg was given, alternating with 400 mg daily; and for those with CrCl < 50 mL/min and hemodialysis patients, 200 mg was given daily. For patients with baseline Hgb > 12 g, no dose adjustment was made, and RBV was discontinued if Hgb.

### 2.5. Assessment of Treatment Efficacy

Sustained virologic response (SVR12) was evaluated by quantitative HCV-RNA PCR at twelve weeks after the end of treatment.

### 2.6. Sample Size Calculation

To evaluate the difference between treatment groups (INF and SOF) and subgroups (INF; INF24, INF48, and SOF; SOF/SIM and SOF/DAC), two-way analysis of variance was performed. A total sample size of 181 was deemed sufficient to detect an effect size of 0.295 at a power of 0.95 (95%) at a partial eta squared of 0.08 at a significance level of 0.05. Taking into account a non-response rate of 10.0%, the sample size was increased to 199 individuals, i.e., 200 patients were applied. Sample size was calculated using power analysis using G*power version 3.9.1.6 for Mac OS.

### 2.7. Statistical Analysis

All collected data were tabulated and statistically analyzed using the following statistical tests. Descriptive data analysis was in the form of percentages, and mean and data were expressed as mean± standard deviation (SD) or number and percentages (%) as appropriate. The Pearson chi-square test or Fisher’s exact test were used for group comparisons of categorical variables. Two-sample *t*-tests were used for all independent variables for numerical data. For categorical data, all independent factors were subjected to univariate binary logistic regression analysis, and the odds ratio with 95% confidence intervals was computed for VR evaluation. All statistical analyses were performed using the computer program SPSS software for windows version 26.0 (Statistical Package for Social Science, IBM Corp, Armonk, NY, USA). A two-tailed *p*-value < 0.05 was considered statistically significant.

## 3. Results

### 3.1. Baseline Characteristics of the Studied Population

The study included a total of 200 chronic hepatitis C patients with complete SVR12 data. Of these patients, 119 (59.5%) were treatment-naïve, and 81 (40.5%) were treatment-experienced. Sixty-one patients received interferon/ribavirin therapy (24 to 48 weeks), 11 patients received sofosbuvir/simeprevir therapy (12 weeks), and 9 patients received sofosbuvir/daclatasvir therapy (12 weeks). The evaluated patients were 18 to 95 years of age (mean ± SD 53.17 ± 15.71 years). Of the 200 patients studied, 119 (59.5%) were male, and 81 (40.5%) were female. Liver cirrhosis was diagnosed in 70 patients (35%), and all cirrhotic patients were compensated; 44 patients had a Child–Pugh score of B, and 24 patients had a Child–Pugh score of A. The main FibroScan score of the studied patients was 8.7 ± 4.1 kPa. Regarding associated comorbidities, most of our patients were overweight, with a mean body mass index of 28.8 ± 6.2. Approximately one-third of our patients were hypertensive, and approximately 39% had been diagnosed with diabetes; 10% had a history of coronary artery disease, and chronic kidney disease had been diagnosed in 23 patients (11.5%). The baseline viral load of the studied patients was 2,340,000 ± 2,150,000 IU/mL. HCV GT4 was the most frequent genotype detected (109 patients; 54,5%) among the infected patients, followed by GT1a and b (64 patients; 32%), GT1b, GT3 (14 patients; 7%), and GT 2 (3 patients; 1.5%). A mixed genotype (GT4 + GT1a) was detected in 10 patients (5%) (Table 1). No statistically significant difference was found among the different genotypes regarding the study patients’ baseline characteristics and prescribed treatment regimens except for gender (*p* = 0.001) (Table 2).

### 3.2. Prescribed Treatment Regimens 

The majority of the study patients (158; 79%) received sofosbuvir-based regimens. Sofosbuvir/daclatasvir with or without ribavirin (SOF/DCV ± RBV) was prescribed for 94 patients (47%), and sofosbuvir/ledipasvir with or without ribavirin (SOF/LDV ± RBV) was prescribed for 64 patients (32%). Other prescribed treatment regimens included ombitasvir/paritaprevir/ritonavir/dasabuvir with or without ribavirin (OBV/PTV/Rtv/DSV ± RBV) (28 patients; 14%), elbasvir/grazoprevir (EBR/GZR) (10 patients; 5%), and glecaprevir/pibrentasvir/ribavirin (GLE/PIB/RBV) (4 patients; 2%). Overall, RBV was included in the treatment regimens for 67 patients (33.5%) (see Table 1). A total of 162 patients (81%) received therapy for 12 weeks, and the remaining 38 patients (19%) were treated for 24 weeks.

### 3.3. Treatment Efficacy

Overall, an SVR12 was attained in 195 patients (97.5%). As shown in Table 3, in terms of the baseline demographic and clinical characteristics, there was no statistically significant difference between the patients who achieved an SVR12 (SVR12 group) and those who did not achieve an SVR12 (non-SVR12 group) apart from the rate of cirrhosis (*p* = 0.032) and the distribution of HCV genotypes (*p* = 0.001). Similarly, the two groups did not differ in terms of the prescribed antiviral drugs (*p* = 0.348 and *p* = 0.755 for the antiviral drug combinations and concomitant RBV therapy, respectively).

**Figure 1:**
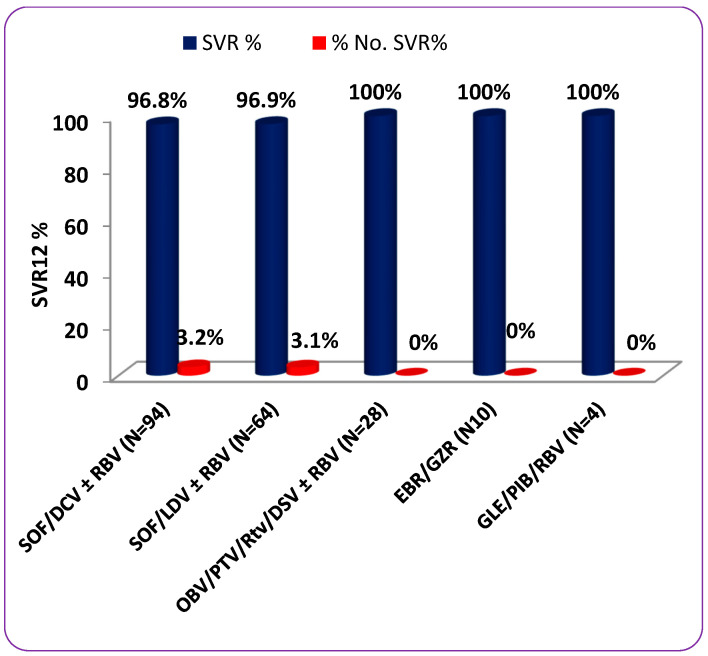
SVR percentage rates stratified by different patients’ characteristics and prescribed drug regimens.

The SVR12 rates were similar among patients in different age groups (100% for patients younger than 40 years, 95.8% for patients 40 to 60 years, and 98.4% for patients older than 60 years; *p =* 0.632), among male and female patients (99.2% versus 95.1%, respectively; *p =* 0.160), or among patients with and without cirrhosis (94.3% versus 99.2%, respectively; *p* = 0.121). In addition, the SVR12 rates did not differ according to the infecting HCV genotype (*p* = 0.847); the percentages were 95.5% for GT1b, 96.3% for GT4, and 100% for GT 1a, GT2, and GT3 and the mixed genotype. On the other hand, a statistically significant difference in SVR12 rates was found among treatment-naïve and treatment-experienced patients (95.8% versus 100%, respectively; *p =* 0.018) (Table 4). Regarding the different drug regimens, there was no statistically significant difference in SVR12 rates achieved by them (*p* = 0.348); the SVR12 rates for SOF/DCV with or without RBV, SOF/LDV with or without RBV, OBV/PTV/Rtv/DSV with or without RBV, EBR/GZR, and GLE/PIB/RBV were 96.8%, 96.9%, 100%, 100%, and 100%, respectively (see Table 4 and Figure 1). In patients receiving SOF/DCV with or without RBV, the SVR12 rates were similar among the patients in different age groups (*p =* 0.898), among male and female patients (*p =* 0.553), among treatment-naïve and treatment-experienced patients (*p* = 0.116), among patients with and without cirrhosis (*p =* 0.252), and among patients infected with different genotypes (*p =* 0.660). Similarly, the efficacy of SOF/LDV with or without RBV did not differ according to age (*p =* 0.878), gender (*p =* 0.613), treatment history (*p =* 0.075), cirrhosis status (*p =* 0.283), or infecting HCV genotype (*p =* 0.448) (see Table 4).

### 3.4. Predictors of SVR12

The patient’s age and gender, treatment history, presence of liver cirrhosis or chronic kidney disease, genotype, and antiviral drug regimen were assessed as potential predictors of the SVR12. Results of the univariate binary logistic regression analysis revealed a nonsignificant effect (*p* > 0.05) of all of the explanatory variables on the outcome. Furthermore, the multivariate binary logistic regression analysis showed a nonsignificant effect (*p* > 0.05) of all of the explanatory variables on the outcome except for gender and the presence of cirrhosis (*p =* 0.045 and *p =* 0.036, respectively) (Table 5). 

## 4. Discussion

Hepatitis C virus infection is a serious challenge to global health with a significant economic impact. Chronic HCV infection is one of the main causes of liver cirrhosis, liver cell failure, and hepatocellular carcinoma. It is the most common indication for liver transplantation worldwide [33,34]. The prevalence of positive HCV antibodies in Saudi Arabia is approximately 0.7%, and the most prevalent genotype is GT4, followed by GT1 [17,35].

Prior to 2011, pegylated interferon alpha and RBV were the recommended antiviral treatments for 24 to 48 weeks. This regimen resulted in a moderate SVR and was associated with multiple side effects [36]. The introduction of the DAA-based regimens achieved high cure rates with minimal adverse effects, better tolerability, and shorter treatment duration [22,28]. Further, treatment with DAA in patients with chronic HCV infection has a positive effect on the bioelectrical brain activity, with an increase in the amplitude of evoked potentials indicating an improvement in the activity of the cerebral cortex, and this improvement was correlated with the neuroimaging parameters [29,30,31,32,33,34,35].

Long-term clinical outcomes and the health-related quality of life may increase with SVR achievement because of the decreased risk of liver disease progression [37].

In the current investigation, we monitored a total of 200 patients who were treated at a single medical facility and were found to have HCV infections. More than half of our patients had GT4 (54%), followed by GT1(32%) including subtypes a and b; this finding was consistent with the overall genotype prevalence among HCV-infected patients in Saudi Arabia [17,38]. Alarfaj et al. [39] reported in a study carried out in Riyadh, Saudi Arabia, that the most observed genotype was GT4 (63.7%), followed by GT1 (24%). According to Bawazer et al. [17], GT4 was the most prevalent genotype in Saudi Arabia, representing 65% of infections, followed by GT1 in 23% of cases. Younger age groups showed an apparent reduction in the prevalence of GT4 but had an increased rate of GT1. Moreover, several review publications and meta-analyses on the distribution of HCV genotypes reported that GT4 predominated in Middle Eastern nations, notably Egypt, Iraq, Saudi Arabia, and Syria, with rates of 86%, 60%, 56%, and 57%, respectively [40,41]. In contrast to our findings, a recent study from Bahrain revealed an increasing tendency of HCV GT1 compared with GT4 in the studied population [29].

Patients in the current study achieved high rates of SVR12 (97.5%), with therapeutic failure occurring only in five cases (2.5%). The high success rates in this study were supported by the results of Alarfaj et al. [39], who reported an SVR12 rate of 95.9% in Saudi Arabia, which is consistent with real-world data reported in Middle Eastern countries [41,42,43].

There was no statistically significant difference in SVR12 rates between age groups, genders, and individuals with or without chronic renal disease, liver cirrhosis, or genotypes. However, there was a statistically significant difference in SVR12 rates between treatment-naïve and treatment-experienced individuals (*p* = 0.018). These findings were consistent with those of Yang et al. [44], who reported no statistically significant variations in SVR rates in patients with various genotypes. These results were also in agreement with the findings of Kamal et al. [45], who studied the effectiveness of DAA-based regimens in elderly Egyptian patients with chronic HCV infection and found that all of these regimens were well-tolerated, safe, and highly successful even in patients aged 75 years or beyond. They found that age did not influence the effectiveness of DAA treatment. 

Regarding patients with chronic kidney disease, the results of this study were in concordance with previous studies in the literature [46,47]; they found that treatment with OBV/PTV/R and DSV with or without RBV was safe and effective in HCV-infected patients with chronic renal disease.

There was no statistically significant difference in SVR12 rates between patients treated with SOF-based regimens and those treated with other regimens regardless of whether they also used RBV. This was in line with previous research and real-world studies from all over the world, showing similar outcomes with high SVRs across a wide range of regimens, genotypes, and durations of therapy with or without the addition of RBV and in both treatment-naïve and treatment-experienced patients [29,30,31,32,33,34,35,36,37,38,39,40,41,42,43,44,45,46,47,48]. Additionally, the previous use of an antiviral medication by the patient did not affect the SVR rate. Therefore, based on these findings, one can say that the proper DAA-based regimens can yield good curative outcomes.

When looking at the patient’s age and gender, whether patients were treatment experienced or treatment-naïve, whether patients had chronic kidney disease or not, and the treatment regimens used and whether RBV was included in them or not, the current study was unable to show a significant difference between the SVR12 group and non-SVR group. However, there were significant differences between the SRV12 and non-SVR12 groups in terms of genotype groups, the presence of liver cirrhosis, and the Child–Pugh score of cirrhotic patients (*p* = 0.032, *p* = 0.02, and *p* = 0.001, respectively). Patients with cirrhosis and a Child–Pugh score of B and GT4 and GT1b had significantly lower SVR12 rates compared with patients without cirrhosis, a Child–Pugh score of A, and viral genotypes other than GT4 and GT1b, according to the results of univariate and multivariate binary logistic regression analyses based on the determinants of the SVR12 (*p* = 0.036). In our research, the presence of cirrhosis and a Child–Pugh score of B stood out as significant predictors of the failure to achieve an SVR12. SOF with RBV treatment resulted in an SVR in only 71.2% of patients with HCV-related cirrhosis, and more than 5% of the patients discontinued the medication owing to side effects; this was consistent with recent Egyptian research that included a large number of patients [49]. Additionally, the findings indicated a gender difference that was statistically significant (*p* = 0.045). In general, SVR12-associated factors were inconsistent between clinical trials and real-world investigations, making it difficult to compare the efficacies of different DAA combinations. Thus far, baseline factors (i.e., liver cirrhosis, past treatment experience, infecting HCV genotype, high viral load, increased liver enzymes, and natural polymorphisms in nonstructural HCV genes that limit drug sensitivity) have been linked to poorer SVR rates [50]. 

The limitation of this study was the limited number of cases (i.e., only 200 cases) compared with the prevalence rate of 1.2% in the overall population of Saudi Arabia. Furthermore, the investigation was carried out at a single center. Additionally, there was no randomization in the patient assignment to treatment [51,52]. This was also confounded by the fact that there was great heterogeneity in the regimens used. Patients younger than 18 years and those with decompensated cirrhosis were not included in the study. 

## 5. Conclusions

The current study verified that DAAs were successful in treating Saudi HCV patients and achieved an SVR in 97.5% of the patients in a real-world context. The presence of cirrhosis, a Child–Pugh score of B, and viral genotypes GT4 and GT1b were significant predictors of the failure to achieve an SVR12.

## Figures and Tables

**Table 1:** Baseline characteristics of the study patients and the prescribed antiviral drugs.

Variable	Study Patients (*n* = 200)
**Age (years)**	
Mean ± SD	53.17 ± 15.71
**Gender**	
Male	119 (59.5)
Female	81 (40.5)
**Treatment history**	
Naïve	119 (60.0)
Experienced	81 (41.54)
INF/RBV (48 weeks)	26 (13)
INF/RBV (24 weeks)	35 (17.5)
SOF/SIM (12 weeks)	11 (5.5)
SOF/DAC (12 weeks)	9 (4.5)
Baseline viral load IU/mL	2,340,000 ± 2,150,000
Body mass index (mean ± SD)	28.8 ± 6.2
FibroScan (mean ± SD)	8.7 ± 4.1
**Fibrosis *n*, (%)**	
F0–F3	130 (70.0)
F4 (Cirrhosis)	70 (35.0)
**Child–Pugh *n*, (%)**	
A	26 (13)
B	44 (22)
**Associated comorbidities (*n*/%)**	
CKD	23 (11.5)
HTN	57 (28.5)
DM	78 (39.5)
IHD	20 (10.0)
None	65 (32.5)
**Baseline lab**	
Albumin (g/dL) mean ± SD (range)	3.47 ± 0.48 (2.9–4.7)
ALT (U/L) mean ± SD (range)	35.48 ± 17.51 (11–71)
Total bilirubin (mg/dL) mean ± SD (range)	1.67 ± 2.62 (0.9–2.7)
Creatinine (mg/dL) mean ± SD (range)	1.07 ± 0.20 (0.5–2.4)
**HCV genotype**	
1(a and b)	64 (32.0)
2	3 (1.5)
3	14 (7.0)
4	109 (54.5)
Mixed (1a+4)	10 (5.0)
**Antiviral drug combinations**	
SOF/DCV ± RBV	94 (47.0)
SOF/LDV ± RBV	64 (32.0)
OBV/PTV/Rtv/DSV ± RBV	28 (14.0)
EBR/GZR	10 (5.0)
GLE/PIB/RBV	4 (2.0)
**RBV included in therapy**	67 (33.5)

**IFN,** Interferon; **RBV**, Ribavirin; **SOF**, Sofosbuvir; **DCV,** Daclatasvir; **SMV**, Simeprevir; **CKD**, chronic kidney disease; **HTN,** hypertension; **DM**, diabetes mellitus; **ALT**, alanine aminotransferase; **LDV**, Ledipasvir; **OBV/PTV/Rtv/DSV**, Ombitasvir, Paritaprevir, Ritonavir, and Dasabuvir; **EBR/GZR,** Elbasvir/Grazoprevir; **GLE/PIB**, Glecaprevir/Pibrentasvir.

**Table 2:** The study patients were stratified by the infecting HCV genotype (baseline characteristics of the study patients and prescribed antiviral drugs based on the infecting HCV genotype).

Study Patients (N)	GT4 (N = 109)	GT1a (N = 42)	GT1b (N = 22)	GT3 (N = 14)	GT2 (N = 3)	Mixed Genotypes (N = 10)	*p*-Value
**Age (years)**							
Mean ± SD	47.43 ± 11.8	48.52 ± 11.9	54.62 ± 19.6	54.82 ± 16.7	55.66 ± 5.8	55.20 ± 15.7	**0.220**
**Gender**							
Male	55 (50.5)	35 (83.3)	13 (59.1)	12 (85.7)	0 (0.0%)	4 (40.0)	0.001 *
Female	54 (49.5)	7 (16.7)	9 (40.9)	2 (14.3)	3 (100.0)	6 (60.0)	
**Treatment history**							
Naïve	62 (56.9)	27 (64.3)	13 (59.1)	9 (64.3)	2 (75)	6 (60.0)	**0.869**
Experienced	47 (43.1)	15 (35.7)	9 (40.9)	5 (35.7)	1 (25.0)	4 (40.0)	
**Cirrhosis status**							
Cirrhotic	39 (35.8)	13 (41.9)	13 (59.1)	2 (14.3)	1 (25.0)	2 (20.0)	**0.075**
Non-cirrhotic	70 (64.2)	29 (69.0)	9 (40.9)	12 (85.7)	2 (75.0)	8 (80.0)	
**Chronic kidney disease**							
Yes	11 (10.1)	9 (21.42)	0	1 (7.14)	0	2 (20)	**0.195**
No	98 (89.91)	33 (78.57)	22 (100)	13 (92.86)	3 (100)	8 (80)	
**Antiviral drugs combination**							
SOF/DCV ± RBV	47 (43.1)	20 (47.6)	9 (40.9)	11 (78.6)	3 (100.0)	4 (40.0)	**0.780**
SOF/LDV ± RBV	38 (34.9)	12 (28.6)	8 (36.4)	2 (14.3)	0	4 (40.0)	
OBV/PTV/Rtv/DSV ± RBV	16 (14.7)	6 (14.3)	5 (22.7)	0	0	1 (10.0)	
EBR/GZR	7 (6.4)	2 (4.8)	0	0	0	1 (10.0)	
GLE/PIB/RBV	1 (0.9)	2 (4.8)	0	1 (7.1)	0	0	
**Ribavirin included in therapy**							
Yes	35 (32.1)	14 (33.3)	12 (54.5)	4 (28.6)	1 (33.3)	1 (10.0)	**0.131**
No	74 (67.9)	28 (66.7)	10 (45.5)	10 (71.4)	2 (66.7)	9 (10)	

* means significant difference. **GT**, genotype; **RBV**, Ribavirin; **SOF**, Sofosbuvir; **DCV**, Daclatasvir; **LDV**, Ledipasvir; **OBV**/**PTV**/**Rtv**/**DSV**, Ombitasvir, Paritaprevir, Ritonavir, and Dasabuvir; **EBR**/**GZR**, Elbasvir/Grazoprevir; **GLE**/**PIB**, Glecaprevir/Pibrentasvir.

**Table 3:** Baseline characteristics of the study patients and the prescribed antiviral drugs according to the SVR12.

Variable	SVR12 Group (*n* = 195)	Non-SVR12 Group (*n* = 5)	*p*-Value
**Age (years)**			
Mean ± SD	52.94 ± 15.79	62 ± 9.03	**0.204**
**Gender**			
Male	118 (60.5)	1 (20.0%)	**0.068**
Female	77 (39.5)	4 (80.0%)	
**Treatment history**			
Naïve	114 (58.46)	5 (100.0%)	**0.230**
Experienced	81 (41.54)	0 (0.0%)	
**Liver cirrhosis**			
Cirrhotic	66 (33.8%)	4(80.0%)	0.032 **
Non-cirrhotic	129 (66.2%)	1 (20.0%)	
**Child–Pugh *n*, (%)**			
A	25 (12.8%)	1 (20.0%)	0.002 **
B	40 (20.5%)	4 (80.0%)	
**CKD**			
Yes	23 (11.79%)	0	**0.716**
No	172 (88.21%)	5 (100%)	
**HCV genotype**			
1a (a and b)	63 (32.3%)	0	0.001 **
2	3 (1.54%)	0	
3	14 (7.18%)	0	
4	105 (53.85%)	4 (80.0%)	
Mixed (1a+4)	10 (5.13%)	0	
**Antiviral drug combinations**			
SOF/DCV ± RBV	91 (46.67%)	3 (60.0%)	**0.348**
SOF/LDV ± RBV	62 (31.79%)	2 (40.0%)	
OBV/PTV/Rtv/DSV ± RBV	28 (14.36)	0	
EBR/GZR	10 (5.13%)	0	
GLE/PIB/RBV	4 (2.05%)	0	
**RBV included in therapy**			
Yes	65 (33.33)	2 (40.0)	**0.755**
No	130 (66.6)	3 (60.0)	

** means significant difference; **SVR12**, sustained virologic response.

**Table 4:** SVR rates stratified by different patients’ characteristics and prescribed drug regimens.

Variable	Overall Patients (N = 200)	SOF/DCV ± RBV (N = 94)	SOF/LDV ± RBV (N = 64)	OBV/PTV/Rtv/DSV ± RBV (N = 28)	EBR/GZR (N = 10)	GLE/PIB/RBV (N = 4)
**Overall patients**	195/200 (97.5%)	91/94 (96.8%)	62/64 (96.9%)	28/28 (100%)	10/10 (100%)	4/4 (100%)
**Age (years)**	***p* = 0.632**	***p* = 0.898**	***p* = 0.878**			
<40	43/43 (100%)	24/24 (100%)	13/13 (100%)	5/5 (100%)	1/1 (100%)	-
40–60	92/96 (95.8%)	45/48 (93.8%)	24/25 (96%)	16/16 (100%)	4/4 (100%)	3/3 (100%)
>60	60/61 (98.4%)	22/22 (100%)	25/26 (96.2%)	7/7 (100%)	5/5 (100%)	1/1 (100%)
**Gender**	***p* = 0.160**	***p* = 0.553**	***p* = 0.613**			
Male	118/119 (99.2%)	58/59 (98.3%)	32/32 (100%)	18/18 (100%)	8/8 (100%)	2/2 (100%)
Female	77/81 (95.1%)	33/35 (94.3%)	30/32 (93.8%)	10/10 (100%)	2/2 (100%)	2/2 (100%)
**Treatment history**	*p* = 0.018 **	***p* = 0.116**	***p* = 0.075**			
Naïve	114/119 (95.8%)	53/56 (94.6%)	38/40 (95%)	12/12 (100%)	8/8 (100%)	3/3 (100%)
Experienced	81/81 (100%)	38/38 (100%)	24/24 (100%)	16/16 (100%)	2/2 (100%)	1/1 (100%)
**Liver cirrhosis**	***p* = 0.121**	***p* = 0.252**	***p* = 0.283**			
Yes	66/70 (94.3%)	29/31 (93.5%)	21/23 (91.3%)	11/11 (100%)	4/4 (100%)	1/1 (100%)
No	129/130 (99.2%)	62/63 (98.4%)	41/41 (100%)	17/17 (100%)	6/6 (100%)	3/3 (100%)
**Genotype**	***p* = 0.847**	***p* = 0.660**	***p* = 0.448**			
1 (a and b)	63/64 (98.4%)	28/29 (96.6%)	20/20 (100%)	11/11 (100%)	2/2 (100%)	2/2 (100%)
2	3/3 (100%)	3/3 (100%)	-	-	-	-
3	14/14 (100%)	11/11 (100%)	2/2 (100%)	-	-	1/1 (100%)
4	105/109 (96.3%)	45/47 (95.7%)	36/38 (94.7%)	16/16 (100%)	7/7 (100%)	1/1 (100%)
Mixed (1a+4)	10/10 (100%)	4/4 (100%)	4/4 (100%)	1/1 (100%)	1/1 (100%)	-

** means significant difference.

**Table 5:** Univariate and multivariate logistic regression analysis for predictors of SVR12.

Predictor	Unadjusted		Adjusted	
	Univariate OR (95% CI)	*p*-Value	Multivariate OR (95% CI)	*p*-Value
**Age (continuous, years)**	**0.962 (0.906–1.021)**	0.205		
**Gender**				
Female	**Reference**		**Reference**	
Male	**6.13 (0.672–55.88)**	0.108	**12.34 (1.056–144.36)**	0.045 **
**Treatment history**				
Naïve	**Reference**		**Reference**	
Experienced	**-**	0.97	**Overestimate**	
**Cirrhosis status**				
No	**Reference**		**Reference**	
Yes	**0.128 (0.014–1.167)**	0.068	**0.071 (0.006–0.843)**	0.036 **
**Antiviral drugs**				
SOF/DCV ± RBV	**Reference**		**Reference**	
SOF/LDV ± RBV	**0.44 (0.02–8.07)**	0.520	**Overestimate**	-
OBV/PTV/Rtv/DSV ± RBV	**6.23 (0.358–107.31)**	0.240	**Overestimate**	-
EBR/GZR	**9.10 (0.58–156.95)**	0.129	**Overestimate**	-
GLE/PIB/RBV	**2.30 (0.160–49.11)**	0.521	**Overestimate**	-
**CKD**				
No	**Reference**		**Reference**	
Yes	**Overestimate**	**-**	**Overestimate**	-

** means significant.
